# Advances and Challenges in Wearable Glaucoma Diagnostics and Therapeutics

**DOI:** 10.3390/bioengineering11020138

**Published:** 2024-01-30

**Authors:** Ryan Shean, Ning Yu, Sourish Guntipally, Van Nguyen, Ximin He, Sidi Duan, Kimberly Gokoffski, Yangzhi Zhu, Benjamin Xu

**Affiliations:** 1 Keck School of Medicine, University of Southern California, 1975 Zonal Avenue, Los Angeles, CA 90033, USA; rshean@usc.edu; 2 Department of Chemical Engineering, Stanford University, Stanford, CA 94305, USA; ning.yu@email.ucr.edu; 3 Terasaki Institute for Biomedical Innovation, 21100 Erwin Street, Los Angeles, CA 90064, USA; s.guntipally@yahoo.com (S.G.); yzhu@terasaki.org (Y.Z.); 4 Roski Eye Institute, Keck School of Medicine, University of Southern California, 1450 San Pablo Street, Los Angeles, CA 90033, USA; van.nguyen2@med.usc.edu (V.N.); kimberly.gokoffski@med.usc.edu (K.G.); 5 Department of Materials Science and Engineering, University of California, 410 Westwood Plaza, Los Angeles, CA 90095, USA; ximinhe@ucla.edu (X.H.); sididuan@ucla.edu (S.D.)

**Keywords:** glaucoma, theranostics, diagnostics, therapeutics, smart contact lens

## Abstract

Glaucoma is a leading cause of irreversible blindness, and early detection and treatment are crucial for preventing vision loss. This review aims to provide an overview of current diagnostic and treatment standards, recent medical and technological advances, and current challenges and future outlook for wearable glaucoma diagnostics and therapeutics. Conventional diagnostic techniques, including the rebound tonometer and Goldmann Applanation Tonometer, provide reliable intraocular pressure (IOP) measurement data at single-interval visits. The Sensimed Triggerfish and other emerging contact lenses provide continuous IOP tracking, which can improve diagnostic IOP monitoring for glaucoma. Conventional therapeutic techniques include eye drops and laser therapies, while emerging drug-eluting contact lenses can solve patient noncompliance with eye medications. Theranostic platforms combine diagnostic and therapeutic capabilities into a single device. Advantages of these platforms include real-time monitoring and personalized medication dosing. While there are many challenges to the development of wearable glaucoma diagnostics and therapeutics, wearable technologies hold great potential for enhancing glaucoma management by providing continuous monitoring, improving medication adherence, and reducing the disease burden on patients and healthcare systems. Further research and development of these technologies will be essential to optimizing patient outcomes.

## 1. Introduction

Glaucoma is the leading cause of irreversible blindness worldwide, affecting an estimated 3.5% of people aged from 40 to 80 years globally [1,2]. The majority of glaucoma cases go undetected and untreated, leading to irreversible vision loss and, ultimately, blindness. The World Health Organization estimates that 7.7 million people with glaucoma experience preventable visual impairment or blindness globally [3]. By 2040, the number of people with glaucoma is expected to increase to 111.8 million, highlighting the urgent need for innovative diagnostic and therapeutic strategies [1].

Glaucoma is a chronic and slowly progressive disease of the optic nerve that tends to be asymptomatic in its early stages. When left untreated, glaucoma can progress to irreversible tunnel vision or even complete visual field loss [4]. Elevated intraocular pressure (IOP) is a major risk factor for the development and progression of glaucoma. Several landmark clinical trials, including the Ocular Hypertension Treatment Study (OHTS) and the Early Manifest Glaucoma Trial (EMGT), have demonstrated that reducing IOP can delay or prevent the onset of glaucoma in individuals with ocular hypertension or early glaucoma [5,6]. These studies provided evidence for the importance of early treatment and IOP control in the management of glaucoma.

Current diagnostic and therapeutic strategies for glaucoma have limitations. As early-stage glaucoma is often asymptomatic, patients may experience delays in receiving diagnostic tests to detect glaucoma, thereby leading to delays in treatment. Even when patients with early glaucoma are evaluated, IOP fluctuations throughout each day may not be accurately represented by a single measurement in the clinic. Therefore, continuous IOP monitoring could advance our understanding of the relationship between IOP and glaucoma onset or progression [7]. Poor patient adherence to medication regimens is another significant problem that contributes to preventable disease progression and vision loss. Additionally, fluctuations and spikes in IOP are considered detrimental, similar to elevated average IOP. Consequently, both conditions benefit from regular monitoring and prompt intervention [8,9]. Therefore, the development of convenient drug delivery systems for glaucoma management has the potential to significantly improve treatment standards. This review aims to provide an overview of current diagnostic and treatment standards, recent medical and technological advances, and current challenges and future outlook for wearable glaucoma diagnostics and therapeutics.

Advancements in wearable devices for the management of glaucoma mirror broader progress observed across numerous medical fields. A recent review article by Kazanskiy et al. highlighted the rapidly growing demand for wearable devices in monitoring various medical conditions, such as diabetes, heart disease, and hypertension [10]. The worldwide market for wearables was USD 978.86 million in 2022 and is expected to grow to USD 4336.7 million by 2032 [10]. Another recent review by Wu et al. outlined numerous advancements in intraocular pressure biosensor engineering [11]. This review aims to expand upon these articles by focusing on extraocular IOP sensors with integrated drug delivery capabilities. By focusing on diagnostic devices with therapeutic capabilities, we present a forward-looking perspective on theranostics—a convergence of diagnostics and therapeutics in an all-in-one device that could reshape future glaucoma management.

## 2. Diagnostics of Glaucoma

### 2.1. Current Methods for Measuring IOP

#### 2.1.1. Goldmann Applanation Tonometer

**Figure 1:**
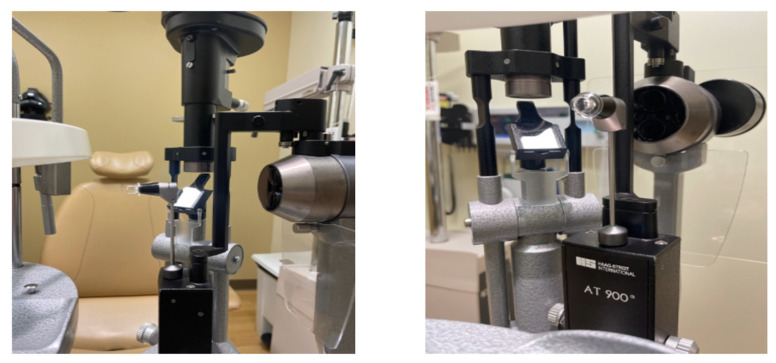
Photographs of a Goldmann Applanation Tonometer.

Goldmann applanation tonometry (GAT) stands as the benchmark for measuring IOP in clinical practice and research and is widely recognized as the gold standard technique [12]. GAT is a slit-lamp-mounted device that uses a calibrated prism to apply a known force to the cornea (Figure 1). The amount of force needed to indent the cornea by a fixed amount is directly proportional to the IOP as long as the cornea has a standard thickness and curvature. GAT has high reproducibility in measuring IOP, demonstrating less variability in IOP measurement than other methods of tonometry [13]. The American Academy of Ophthalmology (AAO) recommends GAT as the standard for IOP measurements in clinical practice. In addition, GAT is the standard in scientific research, including clinical trials to assess the efficacy of glaucoma treatments [14].

The accuracy of GAT measurements can be affected by corneal thickness and curvature, which may lead to an over- or underestimation of IOP [15,16]. Accurate and consistent measurement of IOP with GAT requires a trained operator. GAT involves a patient being seated in a slit-lamp biomicroscope, a setup that may not be practical in situations where patients are bedridden or unable to sit upright. Furthermore, as the tonometer makes direct contact with the cornea, it may cause discomfort for some patients. Additionally, GAT may be affected by ocular surface conditions, including dry eye or corneal edema, which can obscure the true IOP measurement [17]. Finally, as GAT requires specialized equipment and a trained operator, it can only be used in-office and cannot be used to monitor IOP fluctuations continuously.

#### 2.1.2. Handheld Tonometers

A rebound tonometer, an alternative to GAT, is a handheld device that measures the deceleration of a small probe rebounding off of the cornea to calculate IOP. These devices are non-invasive, portable, and can be easily used in clinics or at home, especially by patients who do not tolerate GAT. Rebound tonometers show a high degree of concordance with GAT and yield reproducible IOP measurements [18,19,20]. They also require less patient cooperation and little to no topical anesthesia. A study showed that 73.7% of patients rated rebound tonometry more comfortable than GAT [21]. Therefore, rebound tonometers are well-suited for use with children, elderly patients, and patients with cognitive or physical disabilities who cannot tolerate other IOP measurement methods. Rebound tonometers enable a rapid measuring process, taking just a few seconds per eye, and their compact, portable design allows for use in a wider range of settings compared to GAT.

Similar to GAT, the accuracy of rebound tonometry can be affected by corneal thickness and curvature, overestimating IOP in patients with thicker corneas [22,23,24]. The accuracy and consistency of IOP reading also depend on patient cooperation, blinking during measurements, and corneal hysteresis [25].

**Figure 2:**
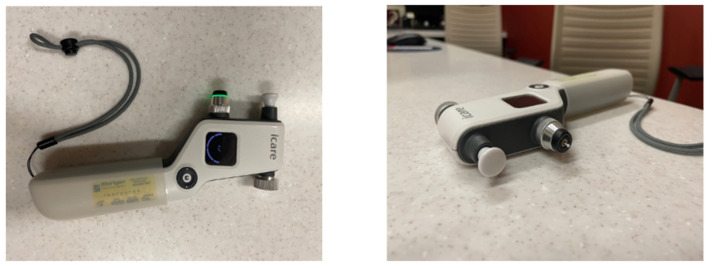
Photographs of an iCare Home Tonometer.

The iCare HOME Tonometer (Figure 2) is a portable rebound tonometer device that allows patients to monitor their IOP levels at home without the need for assistance from a healthcare professional. The iCare HOME Tonometer is an effective tool for monitoring IOP, with 80% of the measurements falling within 3.0 mmHg of IOP measured with GAT [26]. The device is especially useful for patients who have difficulty visiting clinics due to age or mobility, or for those who live in remote areas with limited access to healthcare facilities. The iCare HOME Tonometer can remind patients to check their IOP and alert patients when IOP measurement results are outside of a target range. The users can store and track their IOP data over time using a mobile phone application. Survey results suggest that 78.5% of patients found the iCare Home Tonometer easy to use [27]. The primary limitation of the iCare HOME Tonometer is its cost; the device is relatively expensive and is not covered by most insurances.

#### 2.1.3. Sensimed Triggerfish and GlakoLens

**Figure 3:**
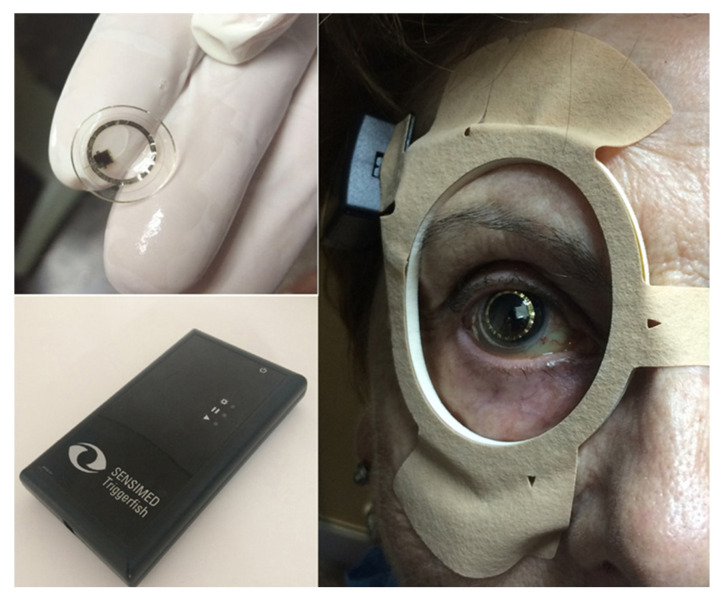
Sensimed Triggerfish contact lens. Adjusted with permission [34]. Copyright 2017, Elsevier.

The Sensimed Triggerfish (Figure 3) is a soft contact lens equipped with a microsensor that continuously measures changes in corneal curvature, which can be used to calculate relative changes in IOP [28]. The device is worn on the eye like a regular contact lens and can be worn during normal daily activities, providing continuous IOP monitoring without the need for manual measurement at specific times. Conventional techniques for measuring IOP, as discussed in Section 2.1, only measure IOP at a single point in time. Therefore, they may not capture diurnal or nocturnal fluctuations in IOP [29]. The Sensimed Triggerfish is the only FDA-approved device for continuous IOP monitoring, offering invaluable data that can enhance the precision of diagnosis and treatment [30,31]. Research about the Triggerfish reported a high level of patient safety and tolerability [32,33].

The Sensimed Triggerfish has several limitations that have hindered its widespread clinical use despite its IOP-monitoring capabilities. First, the Triggerfish measures relative changes in IOP, so another device is still necessary to establish baseline IOP. It can also cause irritation and discomfort because it is an electronic device that is worn on the eye for extended periods of time. Additionally, changes among certain factors, such as air temperature, may add noise to the electronic signal, which can affect the accuracy of intraocular measurements [35,36]. Another major limitation is that the device is expensive but not currently covered by many insurances, which limits its accessibility. Finally, the device cannot be used in patients who are unable to tolerate wearing contact lenses.

In addition to the Triggerfish, the GlakoLens (Istanbul, Turkey) is an IOP-sensing smart contact lens that has not yet received FDA approval. The design of the GlakoLens system involves an electrically passive sensor embedded in a disposable soft contact lens which utilizes resonant frequency shift of a metallic resonator for 24-h IOP monitoring. The sensor’s meta-material properties enable accurate measurement of IOP fluctuations by detecting changes in corneal geometry. Data from the sensor can be collected wirelessly via a low-power RF signal generated by a Holter-monitor-like device with an electronic circuit and wearable antenna. GlakoLens has taken proactive steps at commercialization, developing a user-friendly website and intellectual property protection (US Patent No: US10067075B2 and pending PCT applications). By prioritizing ease of use, design innovation, and intellectual property protection, GlakoLens may achieve successful commercialization in the coming years.

### 2.2. Recent Advances in Wearable Diagnostics

Wearable diagnostics are a relatively new development in ophthalmology, offering the ability to monitor IOP using contact lenses and implants. Wearable IOP sensors can measure IOP continuously or at predefined intervals, enabling real-time monitoring and timely detection of IOP fluctuations. In addition to contact lens-based sensors, there has been research into implantable IOP sensors in sites such as the anterior chamber, capsular bags, vitreous, and choroid [37]. Wearable IOP sensors and other diagnostic tools can be used for diagnosing, monitoring, and predicting the progression of glaucoma. Continuous IOP monitoring can provide valuable information on IOP fluctuations, which is an important risk factor for glaucoma progression [37,38]. In addition to IOP, these sensors have the potential to monitor glucose, lactate, and cortisol levels as well as the humidity of the ocular surface, which enables the possibility of monitoring for and detecting additional conditions, such as diabetes and liver disease [37,39,40,41].

**Figure 4:**
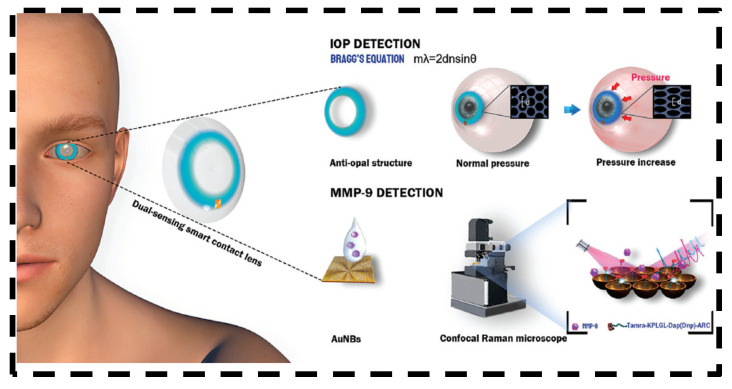
Schematic illustration of dual-functional contact lens for IOP detection and MMP-9 detection. Open access [42]. Copyright 2022, Wiley-VCH GmbH.

Wang et al. developed a smart contact lens with a dual-sensing platform for real-time monitoring of IOP and detecting matrix metalloproteinase-9 (MMP-9) in tears, which is a biomarker in eye-related diseases including glaucoma [42]. The unique design (Figure 4) contained surface-enhanced Raman spectroscopy (SERS) substrates. IOP could be monitored by observing structural color changes, and glaucoma could be predicted using quantitative SERS measurement of MMP-9 levels in tear film. Tests using porcine eyes found that the contact lens provided accurate measurements of IOP in the range from 0 to 30 mmHg (R2 = 0.98), covering the normal physiological IOP range (10–20 mmHg). Meanwhile, the SERS analysis can effectively detect MMP-9 down to concentrations of 1.29 ng/mL.

**Figure 5:**
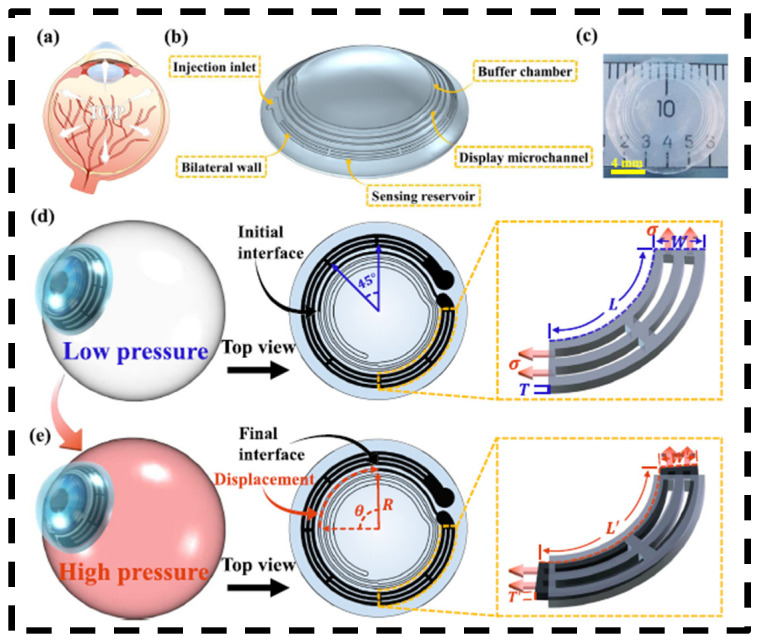
Schematic diagrams, photograph, and operation principle of the microfluidic contact lens. (**a**) Schematic diagram of the human eye with the contact lens. (**b**) Schematic diagram of the contact lens. (**c**) Photograph of the contact lens. (**d**,**e**) The operation principle of the contact lens in the setting of elevated IOP. The mechanical mechanism of the sensor is represented on the right. Adapted with permission [44]. Copyright 2023, Elsevier.

Electronic smart contact lenses that monitor IOP changes usually have relatively simple structural designs and can be constructed from easily accessible materials. However, these devices sometimes have difficulty differentiating small changes in IOP from signal noise induced by activities such as blinking. In order to reduce noise, Hu et al. created a contact lens with a self-lubricating layer that reduces the coefficient of friction to remove interference from the tangential forces [43]. The contact lens is essentially composed of three distinct layers (Figure 5), including a substrate layer, a flexible reinforced sensing layer, and a self-lubricating layer. The IOP sensor maintains the same level of sensitivity to normal forces with the addition of the self-lubricating layer, while significantly reducing the effects induced by blinking and eye movement.

While electronic wearable devices can provide consistent real-time IOP monitoring, the inclusion of electronic components in contact lenses reduces water permeability and may cause corneal damage with long-term use. A structurally colored contact lens that changes color in response to changes in IOP can effectively avoid this problem. These contact lenses are composed of flexible materials and are free of complex electronic components [45,46,47,48]. Chen et al. designed a high-sensitivity microfluidic contact lens sensor composed of a sensing reservoir filled with dyed fish oil, a display microchannel, and a buffer chamber [43]. When IOP increases, the corneal radius of curvature increases and the enlarged volume of the sensing reservoir under the surface tension of the tear film pushes liquid in the display microchannel into the sensing reservoir. The displacement of the liquid interface reflects the IOP change. The contact lens can reach a sensitivity of 660 μm/mmHg in the IOP fluctuation range of 10–30 mmHg with a linear regression coefficient R2 up to 0.99. However, one limitation of this device is that small IOP changes are indistinguishable by the naked eye and are only detectable with specialized reflection spectrometers.

**Figure 6:**
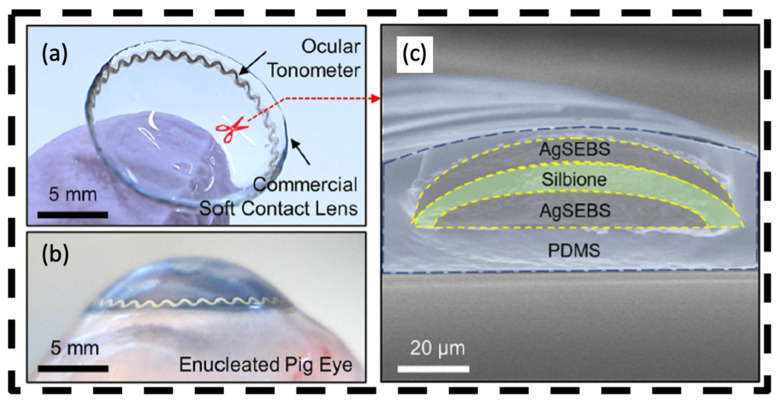
Schematic and optical images of smart soft contact lens for 24 h IOP monitoring. (**a**) Photograph of the contact lens. (**b**) Cross-sectional scanning electron microscope (SEM) image of the contact lens. (**c**) Photograph of the contact lens in an enucleated pig eye. Open access [49]. Copyright 2022, Springer Nature.

Continuous monitoring of IOP during sleep has been challenging in glaucoma care. In an attempt to address this issue, Lee et al. created a smart soft contact lens (SSCL) with an ocular tonometer built into its structure [49]. This device uses a circuit with a resistor, inductor, and capacitor in series (RLC) to produce a distinct electrical resonance frequency based on the characteristics of the inductor and capacitor (Figure 6). The electrical properties of the RLC circuit in this ocular tonometer vary depending on the radial and axial deformations by the contact lens. As a result, the RLC circuit produces a different resonance frequency when IOP increases, followed by curvature changes of both the eye and the contact lens. The device offers overnight wearability, superior signal quality compared to existing wearable ocular tonometers, and comfort on par with the Triggerfish. Additionally, the lens has no internal power source; rather, its IOP measurements can be read using glasses or a sleep mask fitted with a reader coil inductively paired with the ocular tonometer. This SSCL was fabricated to mirror commercial brands, preserving its inherent characteristics such as great biocompatibility, softness, transparency, and oxygen transmissibility.

Variations in oxygen and water content, as well as the stiffness of the contact lenses, may lead to discomfort for certain users and introduce signal noise. To address these issues, Zolfaghari et al. created wearable glasses with a laser source, lenses, mirrors, mask, and a camera for personalized real-time IOP monitoring [50]. Using the lens, mirrors, and mask, it creates a grid on the cornea which it measures with the camera to detect changes in corneal curvature. This device was justified with analytical modeling, ray tracing, and FEM simulations [50]. It produced a pressure measurement resolution of 2.4 mmHg between 0 and 55 mmHg pressure. Zolfaghari et al. produced a separate glasses-based solution with an implantable diffraction grating MEMS sensor [51]. The readout glasses were embedded with a laser dioxide, miniaturized aspheric lenses, and a complementary-metal-oxide semiconductor (CMOS) camera [51].

While wearable IOP sensors and other glaucoma diagnostics offer several advantages, they also have limitations. Some materials used in contact lens sensors, such as polymerized hydroxyethyl methacrylate (pHEMA) and silicone hydrogel (SiH), may be affected by local hydration levels, which can introduce noise and inaccuracies to IOP measurements [37]. Due to signal noise and variations in corneal parameters, these wearable diagnostics are not as accurate or stable as GAT, which is considered the clinical standard for measuring IOP [14,37]. Furthermore, the cost of wearable glaucoma diagnostics may prevent some patients from accessing them in the future. Further research is needed to establish the efficacy, safety, and cost-effectiveness of wearable glaucoma diagnostics.

## 3. Therapeutics

### 3.1. Current Methods for Lowering IOP

#### 3.1.1. Eye Drops

Eye drops are a common form of glaucoma treatment that can be prescribed in different classes, including prostaglandin analogs, beta blockers, alpha agonists, and carbonic anhydrase inhibitors (CAIs). The primary goal of using eye drops is to prevent or slow down the progression of glaucoma by lowering IOP, either by decreasing the amount of aqueous humor produced by the eye or improving its outflow from the eye [52].

Prostaglandin analogs are the most commonly prescribed eye drops due to their effectiveness in reducing IOP and convenient once-a-day dosing [52]. They work by increasing the outflow of aqueous from the eye via the uveoscleral pathway. They have greater efficacy, lowering IOP by 25 to 30%, and fewer systemic side effects compared to other eye drop medication classes. However, like many typical glaucoma medications, prostaglandin analogs may induce eye redness and other localized side effects in the eye and periocular area, such as pigmentary changes, lengthening of eyelashes, fat atrophy, and an increased risk of uveitis and cystoid macular edema [53].

Latanoprostene bunod, marketed under the name Vyzulta, is a novel glaucoma treatment that combines latanoprost, a prostaglandin analog, with a nitric oxide donor, offering a dual mechanism to reduce IOP [54]. Latanoprost increases uveoscleral outflow, while nitric oxide increases conventional outflow through the trabecular meshwork, resulting in an average IOP reduction of 27% within 24 h with a generally favorable safety profile [55,56]. Beta-blockers reduce IOP by inhibiting aqueous humor production, but they can adversely affect cardiovascular and respiratory systems, potentially reducing lung function and increasing asthma morbidity [57,58,59]. Alpha-adrenergic agonists decrease aqueous humor production and increase outflow, achieving a 20–25% IOP reduction. Despite effectiveness, they may cause allergic conjunctivitis and systemic side effects, making them less common as first-choice monotherapy but widely used in multi-drug glaucoma treatment [60,61].

Carbonic anhydrase inhibitors reduce IOP by decreasing aqueous humor production but are less commonly used as monotherapy due to potential side effects like metallic taste, ocular irritation, and corneal edema [6,62]. Netarsudil, a novel eye drop, lowers IOP by inhibiting Rho kinase and norepinephrine transporter, enhancing trabecular outflow and reducing aqueous production [54]. Clinical studies show a 20–25% IOP reduction, with ocular side effects like conjunctival hyperemia, subconjunctival hemorrhage, and blurred vision, but relatively rare systemic side effects compared to beta-blockers and alpha agonists [54,63].

Despite the benefits of using eye drops as a therapeutic option for glaucoma, there are numerous limitations associated with their use. Eye drops require careful administration and may have side effects such as ocular surface irritation and systemic effects [58,59]. Moreover, some patients may not be able to tolerate the side effects associated with different classes of eye drops, resulting in nonadherence and insufficient IOP management, particularly among those patients with limited health literacy [64]. Finally, patients who have difficulty administering the drops may need support from a caregiver or healthcare professional, potentially leading to financial difficulties and increased noncompliance [65].

#### 3.1.2. Intracameral Implants

Intracameral implants emerge as a promising new approach for treating glaucoma. These are small devices that can be implanted into the eye to deliver medications. Several varieties of intracameral implants, such as DURYSTA (Allergan, Irvine, CA, USA), OTX-TIC (Ocular Therapeutix, Bedford, MA, USA), and iDose (Glaukos, Aliso Viejo, CA, USA), offer direct and sustained medication release into the anterior chamber of the eye. DURYSTA is an intracameral implant that was recently approved by the FDA for the treatment of open-angle glaucoma or ocular hypertension [66]. It is a biodegradable implant that slowly releases bimatoprost, a prostaglandin analog. DURYSTA is the only FDA-approved intracameral implant, providing a sustained reduction in IOP of 20 to 25% [67]. However, DURYSTA is only approved for a single dose due to the risk of corneal edema [66,68]. iDose is another example of an intracameral implant that has demonstrated success in several clinical trials [68]. It is a small titanium implant that is surgically inserted into the trabecular meshwork to deliver a formulation of travoprost over a period of six to twelve months [68]. After all the travoprost has been depleted, this implant can be removed and exchanged [69]. OTX-TIC is another intracameral implant that has shown promising results in clinical trials [68]. Similar to DURYSTA, it is a biodegradable implant that is designed to release a formulation of micronized travoprost over an extended period, typically spanning four to six months. Once the travoprost supply is depleted, the implant is bioresorbable.

The main disadvantage of intracameral implants is that they are invasive and have short-term risks, such as infection or bleeding, and long-term complications, such as endothelial damage and persistent corneal edema. Intracameral implants can also be more expensive than other treatment options [68]. Finally, given that intracameral implants are a relatively recent therapeutic innovation, further research is required to ascertain the safety of repeated injections [70].

#### 3.1.3. Lasers and Surgery

Laser and surgical treatments serve as adjuncts to medications in the comprehensive management of glaucoma. Laser therapies are generally considered low-risk and can decrease aqueous production or enhance aqueous outflow via the trabecular meshwork, efficiently lowering IOP and managing glaucoma. Common laser therapies used to treat glaucoma include trabeculoplasty, peripheral iridotomy, and cyclophotocoagulation [71]. Surgery remains the definitive standard for lowering IOP when glaucoma proves resistant to medical or laser treatment [72,73]. Glaucoma surgeries range from minimally invasive glaucoma surgery (MIGS), offering reduced risk with modest efficacy, to traditional glaucoma surgeries like trabeculectomy and tube shunts, which yield higher efficacy but come with increased risk. Nonetheless, glaucoma surgery carries significant risks, including infection, bleeding, and permanent vision loss [74]. Surgery may also be inaccessible to some patients due to the high costs of surgical treatment.

### 3.2. Recent Advances in Wearable Therapeutics

Drug-eluting contact lenses, soft contact lenses designed to release medication into the eye over an extended period of time, can be used to treat a wide range of ocular conditions [75,76]. These contact lenses offer the advantage of sustained drug release, which improves medication adherence and reduces side effects. Drug-eluting contact lenses can release therapeutic levels of medication for up to several months, demonstrating their potential as a sustained drug delivery system [77]. Some studies have reported that drug-eluting contact lenses result in greater IOP reduction than eye drops [78]. As patient nonadherence to conventional eye drops poses a significant obstacle to effective glaucoma care, drug-eluting contact lenses are seen as a promising alternative treatment option [79].

**Figure 7:**
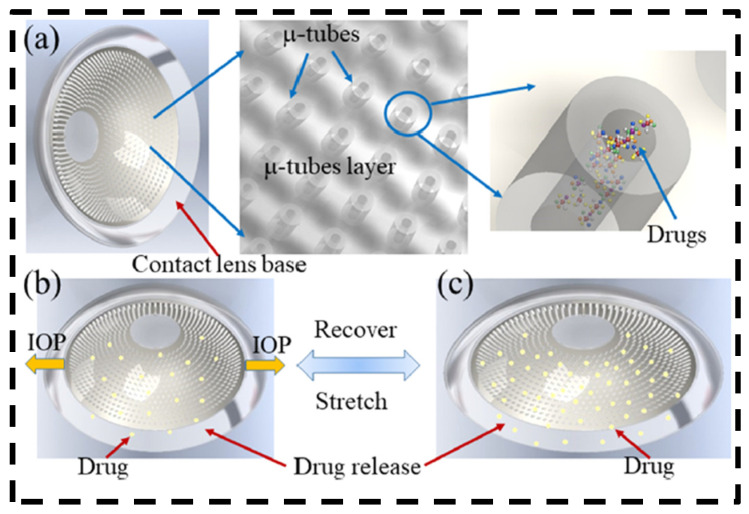
Schematic illustration of contact lens embedded with microtubes as drug containers. (**a**) Schematic diagram of the contact lens with embedded microtubes as drug containers for diffusion-based drug delivery and adaptive drug delivery. (**b**) Schematic of the contact lens device under a neutral state for diffusion-based drug release. (**c**) Illustration of the mechanically stretched contact lens device with more drug being released. Adjusted with permission [80]. Copyright 2020, American Chemical Society.

A novel type of contact lens was recently developed by Que et al. with embedded microtubes as drug containers (Figure 7) [80]. This type of contact lens can be fabricated by combining a ball-mold fabrication process and soft lithography. Drug release is based on diffusion and adaptive mechanisms; when IOP rises, the contact lens is stretched and the deformation of the microtubes triggers the diffusion of drugs. By tuning the tube size, density, and drug loading, the contact lens can achieve an extended drug release of up to 45 days.

While the immersion method is the simplest and most cost-effective way to prepare drug-loaded contact lenses, it has the disadvantages of low drug loading and fast release speed [81,82]. Shiau et al. resolved these issues by incorporating large-pore mesoporous silica nanoparticles (LPMSNs) with high surface area, large pore volume, and tunable pore size into drug-eluting contact lenses (DCLs) [83]. LPMSN-laden DCLs do not require drug preloading and can be fabricated with current contact lens manufacturing processes. Compared with standard DCLs, LPMSN-laden DCLs exhibited enhanced maximum loading and release capacities for glaucoma drugs and slower release rates, significantly extending the duration of drug release.

**Figure 8:**
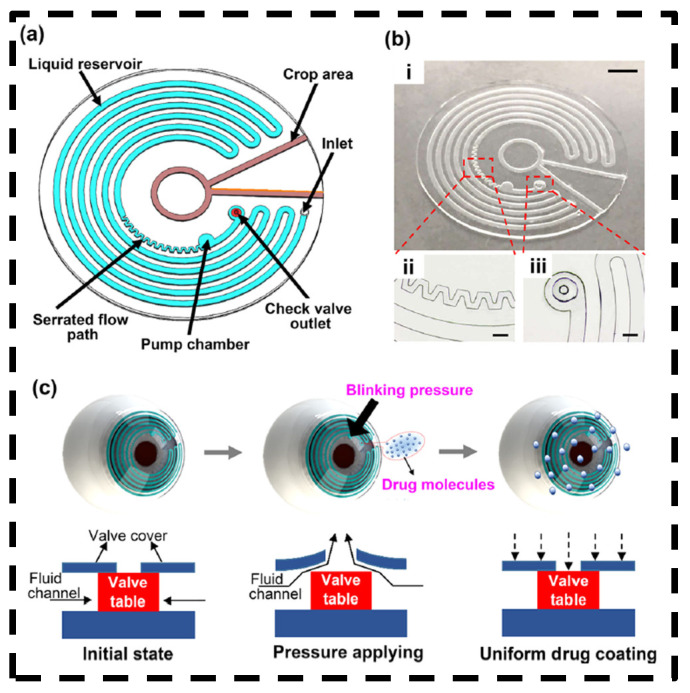
Schematic diagram and work principle of flat microfluidic contact lens. (**a**) Schematic diagram of the microfluidic chip. (**b**) (**i**) Picture of a flat microfluidic chip, scale bar: 2 mm; (**ii**) Enlarged view of the serrated flow path, scale bar: 300 μm; (**iii**) Enlarged view of the check valve outlet, scale bar: 300 μm. (**c**) Schematic illustration of drug release process realized by the pressure-triggered microfluidic contact lens. Adjusted with permission [47]. Copyright 2022, American Chemical Society.

In another study, a type of flat microfluidic contact lens was fabricated, integrating a microchannel and a micropump (Figure 8) [47]. Drugs confined in the microchannels can be released by applying pressure on the pump chamber, such as through blinking, making liquid release controllable and adjustable. Different types of drugs can be loaded in different microchambers for multi-drug treatment applications. The contact lens exhibited good flexibility, light transmission, and biocompatibility without the need for electronic components, providing a safe, convenient, and effective method to treat ocular diseases.

The bimatoprost periocular ring (Allergan, Irvine, CA, USA) is a ring-shaped drug-eluting device that rests under the eyelids in the fornix. The device has passed Phase 2 trials, with the ability to elute bimatoprost and reduce IOP for up to 6 months [84]. Similar to contact lenses, periocular rings allow eye care providers to address the issue of medication nonadherence. However, to date, no periocular rings have received FDA approval for the treatment of glaucoma.

There are currently no FDA-approved drug-eluting contact lenses to treat glaucoma. However, drug-eluting contact lenses have been approved to treat other eye conditions, including myopia [85]. One issue with drug-eluting contact lenses is that they can deliver only small amounts of medication to patients, and the rate of release may not be linear [77]. In addition to potential safety concerns, patient and practitioner acceptance, and storage considerations, fit, comfort, and cost present further challenges [86]. Further research and development are essential to overcome these limitations before the full potential of drug-eluting contact lenses as a wearable therapeutic for glaucoma can be harnessed.

## 4. Emerging Theranostic Platform

### 4.1. Description of Theranostics

Theranostics are a category of medical devices that integrate therapeutic drugs and diagnostic modalities into a united system [87]. Instead of using a “one size fits all” strategy, theragnostic adopts a personalized approach to patient management by customizing care to individual disease profiles and treatment responses [87,88]. Consequently, theranostic devices can automatically modify medication dosages in response to disease condition fluctuations [87]. In the context of glaucoma care, an example of a theranostic device is one that can monitor IOP and use this information to dynamically modulate the release of IOP-lowering drugs. Moreover, theranostic devices have the potential to monitor and image diseased tissue, analyze delivery kinetics, and maximize drug efficacy [87].

The interest in theranostics has grown significantly since the beginning of the century. Between 2000 and 2011, the annual publication count for research on theranostics and multifunctional therapies increased from none to 120 and 160 papers, respectively. The growing interest in theranostics, including dedicated journals on the topic, indicates its rising importance in medicine [89].

### 4.2. Clinical Uses of Theranostics

One of the challenges in glaucoma management is the accurate and continuous monitoring of IOP. As previously mentioned, traditional methods only provide intermittent IOP measurements during clinic visits. However, theranostic smart soft contact lenses (SSCLs) can provide continuous and non-invasive IOP monitoring. These lenses incorporate biosensing components that measure and transmit real-time IOP data wirelessly. By providing a comprehensive understanding of IOP dynamics, theranostic SSCLs facilitate personalized treatment strategies and timely interventions.

Glaucoma often requires long-term medication to control IOP and prevent disease progression. Conventional treatment methods, such as eye drops, have limitations in terms of patient adherence and drug bioavailability [64]. Theranostic SSCLs address these challenges by offering personalized drug delivery directly to the ocular surface. These lenses facilitate the controlled release of therapeutic agents, maintaining consistent drug levels over an extended duration. Theragnostic SSCLs hold the potential to circumvent the drawbacks of eye drops, thereby enhancing medication adherence, improving drug efficacy, and reducing patient discomfort [78]. Moreover, theranostic SSCLs can incorporate feedback mechanisms that optimize treatment based on individual patient needs. By integrating biosensing capabilities with drug delivery systems, these lenses can monitor IOP levels and adjust drug release accordingly. This dynamic approach ensures precise medication administration, minimizing IOP fluctuations and optimizing therapeutic outcomes. Theranostic SSCLs offer a minimally invasive and patient-friendly approach to provide continuous IOP monitoring and personalized drug delivery, which have the potential to revolutionize glaucoma management. By improving the treatment of glaucoma, they may even reduce the number of clinic visits and invasive procedures. Further research is needed to fully explore the capabilities of these technologies and their integration into routine clinical practice.

### 4.3. Emerging Technologies

Recent advances in smart contact lenses for glaucoma diagnosis and drug delivery have inspired growing research interest in the integration of both types of functionalities to enable continuous IOP monitoring and effective on-demand drug delivery to treat glaucoma. This closed-loop feedback system features a sensor in the contact lens that detects elevated IOP and an on-board signal processor that triggers the immediate release of preloaded drugs to lower IOP. When the IOP drops below a predetermined threshold, drug release would then be halted.

**Figure 9:**
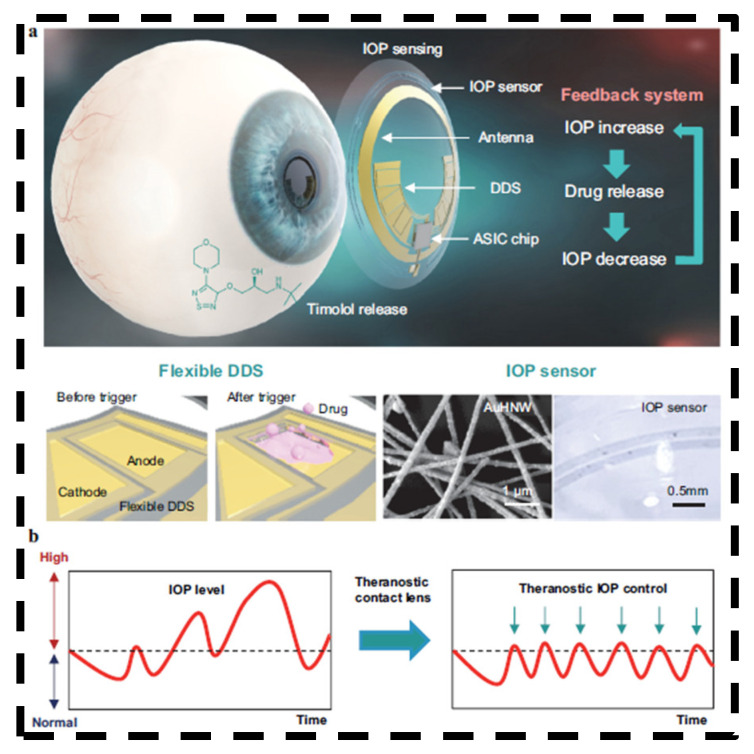
Schematic illustration of AuHNW-based theranostic contact lens for glaucoma treatment. (**a**) The structure of theranostic smart contact lens with a fully integrated AuHNW-based IOP senor, a DDS, and wireless circuits for wireless glaucoma treatments with a feedback system for IOP sensing and timolol release. (**b**) Schematic representation of the conventional continuous IOP monitoring and the IOP control by IOP monitoring and on-demand drug delivery for the treatment of glaucoma. Open access [90]. Copyright 2022, Springer Nature.

Hahn et al. recently developed a theranostic device that integrates electrical circuits on a contact lens for IOP monitoring, wireless data transmission, and coordinated drug delivery (Figure 9) [90]. The key attribute of this lens is its feedback mechanism, equipped with a highly sensitive gold hollow nanowire sensor for real-time IOP monitoring, and an adaptive drug delivery system that releases the glaucoma medication timolol on demand to modulate IOP. The IOP sensor can attain measurements on par with commercial tonometers, with a correlation coefficient of R = 0.94. Another significant benefit is that the technology can be personalized to react to a patient’s unique IOP levels and treatment sensitivity. A wireless board receives the IOP measurements from the lens, which are subsequently transferred to and interpreted by a computer using low-energy Bluetooth.

Xie et al. developed another closed-loop theranostic SSCL that demonstrated IOP sensing and drug delivery capabilities called the wireless theragnostic contact lens (WTCL) [91]. This lens utilizes an iontophoresis drug delivery system, which allows for electrically controlled medication release and improved drug permeation. A double-layer structure was adopted to overcome the challenge of integrating multiple modules onto the space-limited, curved contact lens (Figure 9). Sensors and wireless power transfer circuits were embedded inside the contact lens between the double layers to avoid direct contact with the ocular surface. An ultra-soft air dielectric film between the layers provides high sensitivity to IOP fluctuations. Drug release from the hydrogel layer coated on an iontophoretic electrode is activated when IOP is higher than 21 mmHg. Using iontophoresis drug delivery, the WTCL can achieve an IOP reduction of over 20%, surpassing the 6.9 ± 14.7% lowering seen with slow diffusion over extended periods (~2 h) in rabbits. The WTCL IOP sensor maintains an error rate of <42%, compared to 10–14% error associated with a Tonopen.

## 5. Current Challenges and Clinical Outlook

Smart contact lenses with diagnostic and therapeutic capabilities have the potential to revolutionize glaucoma management by providing continuous monitoring and targeted treatments. Diagnostic lenses offer a non-invasive and user-friendly alternative to traditional diagnostic tools like GAT, enabling the continuous monitoring of IOP fluctuations, facilitating early glaucoma detection and allowing for timely treatment before disease progression. Despite the advancements in the field of diagnostic lenses, current iterations are not without their limitations. Challenges such as the bulkiness and rigidity of embedded circuits, the failure to detect minute fluctuations in IOP, and the complexities involved in their manufacture, persist. Hydrogel-based colorimetric sensors emerge as a promising solution to these issues thanks to their inherent flexibility, straightforward production process, and the elimination of the need for an external power source [92]. These sensors employ various mechanisms for color or transparency alteration, including interference [93], scattering [94], and diffraction [95]. Some mechanisms have shown exceptional sensitivity to even the slightest deformations [96]. The potential for integrating such hydrogel sensors into contact lenses to monitor IOP changes is significant, bringing new possibilities to non-invasive tracking of ocular health. Therapeutic contact lenses can be personalized based on each patient’s specific needs for controlled drug delivery. Advancements in smart contact lens technology could significantly improve patient adherence to glaucoma medications and reduce the need for frequent visits to the doctor’s office for monitoring. The development of closed-loop theranostic contact lenses combines the advantages of diagnostic and therapeutic lenses by simultaneously diagnosing and treating glaucoma.

While the potential benefits of smart contact lenses are exciting, it is important to note that the technology is still in the early stages and numerous challenges remain. First, the integration of sensors, drug delivery, and communication components into a contact lens presents a complex challenge, as ensuring the reliability, stability, and compatibility of all these components is demanding. Second, the complicated designs of the smart contact lenses and their use of electronics could make them uncomfortable to wear and increase their cost of production, which would hinder adoption. In addition, smart contact lenses come into direct contact with the eye; therefore, materials must be safe and biocompatible to avoid irritation and inflammation. Finally, closed-loop smart contact lenses require a power source that is stable and compact enough to operate the sensors, deliver the drugs, and wirelessly communicate with external systems.

While smart contact lenses have the potential to transform glaucoma diagnosis and treatment, their development and commercialization might take several more years due to the aforementioned challenges. As technology continues to advance, smart contact lenses could shift glaucoma practice paradigms by providing a lower-cost, closed-loop theranostic system that addresses the urgent needs of both eye care providers and patients. However, further research, validation, and regulatory clearances are necessary before smart contact lenses can become a standard component of routine glaucoma management.

## Data Availability

Not applicable.
